# Development of a real-time loop-mediated isothermal amplification assay for detection of porcine circovirus 3

**DOI:** 10.1186/s12917-019-2037-z

**Published:** 2019-08-23

**Authors:** Huanan Wang, Xiangnan Liu, Fanwen Zeng, Tongyuan Zhang, Yuexiao Lian, Miaoli Wu, Li Xiao, Yujun Zhu, Yu Zhang, Meili Chen, Ren Huang, Manlin Luo, Feng Cong, Pengju Guo

**Affiliations:** 1 Department of Veterinary Medicine, Zhejiang Provincial Key Laboratory of Preventive Veterinary Medicine and College of Animal Sciences, Zhejiang University, Hangzhou, 310058 China; 2 Guangdong laboratory animals monitoring institute and Guangdong Provincial Key Laboratory of Laboratory Animals, Guangzhou, 510633 China; 3 Guangdong Provincial Key Laboratory of Zoonosis Prevention and Control, College of Veterinary Medicine, South China Agricultural University, Guangzhou, 510640 China; 4 Center for Animal Disease Control and Prevention, FuShun, 113006 China; 5 Zhejiang Provincial Key Laboratory of Preventive Veterinary Medicine, College of Animal Sciences, Zhejiang University, Hangzhou, 310058 China

**Keywords:** Porcine circovirus 3, Real-time LAMP, Epidemiological investigations

## Abstract

**Background:**

Porcine circovirus type 3 (PCV3) is an emerging circovirus species, that has been reported in major pig-raising countries including the United States, China, South Korea, Brazil, Spain, and Poland.

**Results:**

A real-time loop-mediated isothermal amplification (LAMP) assay was developed for rapid detection of porcine circovirus 3 (PCV3). The method had a detection limit of 1 × 10^1^ copies/μL with no cross-reactions with classical swine fever virus (CSFV) C strain, foot-and-mouth disease virus (FMDV), porcine circovirus 2 (PCV2) LG vaccine strain, porcine epidemic diarrhoea virus (PEDV), porcine respiratory and reproductive syndrome virus (PRRSV), or pseudorabies virus (PRV). The PCV3 positive detection rate of 203 clinical samples for the real-time LAMP assay was 89.66% (182/203).

**Conclusions:**

The real-time LAMP assay is highly sensitive, and specific for use in epidemiological investigations of PCV3.

## Background

In June 2015, an outbreak of porcine dermatitis and nephropathy syndrome (PDNS) was reported in a commercial pig farm in North Carolina, United States. Compared with the historical data, the mortality rate of sows increased by 10.2% and the pregnancy rate dropped 0.6%. The affected sows were anorexic and their skin presented multifocal papules, plaques and superficial dermatitis. The manifestation of infected foetuses included weak, stillborn and mummified individuals. A new virus was isolated from these animals with the help of next-generation sequencing technology and was identified as porcine circovirus 3 (PCV3) [1]. PCV3 was subsequently widely detected in China, Korea, Brail and many other European countries including Poland, Italy, Spain, Denmark, Germany, and the United Kingdom [2–6].

Porcine circovirus 3 (PCV3) is a member of the *Circovirus genus* in the *Circoviridae* family. The clinical symptoms and pathological changes of PCV3-infected pigs were highly similar to those of PCV2-infected pigs [7]. Given that the virus may be highly prevalent globally, an accurate laboratory diagnosic for rapid confirmation of PCV3 infection is needed. Recently, conventional loop-mediated isothermal amplification (LAMP) assays and PCR-based diagnostic assays for PCV3 have been developed [8–10]. Real-time LAMP is a constant temperature amplification method carried out at 60–65 °C, for which only a simple water bath is required. Real-time LAMP eliminates reverse transcription steps as well as PCR instrument cooling time, which shortens the amplification time. Adding a fluorescent DNA intercalating dye into the real-time LAMP reaction enables monitoring of a fluorescence amplification curve [11]. Compared to conventional LAMP assays, this method avoids visible error, enables quantitative detection and is more suitable for multi-sample analysis. Because of its simple reaction conditions and ease of use, the real-time LAMP method has been adapted for the detection and diagnosis of a variety of pathogens [12, 13]. In this study, a real time RT-LAMP assay was developed for the rapid diagnosic of PCV3.

## Results

### Sensitivity and specificity of the real-time LAMP assay for rapid detection of PCV3

**Fig. 1:**
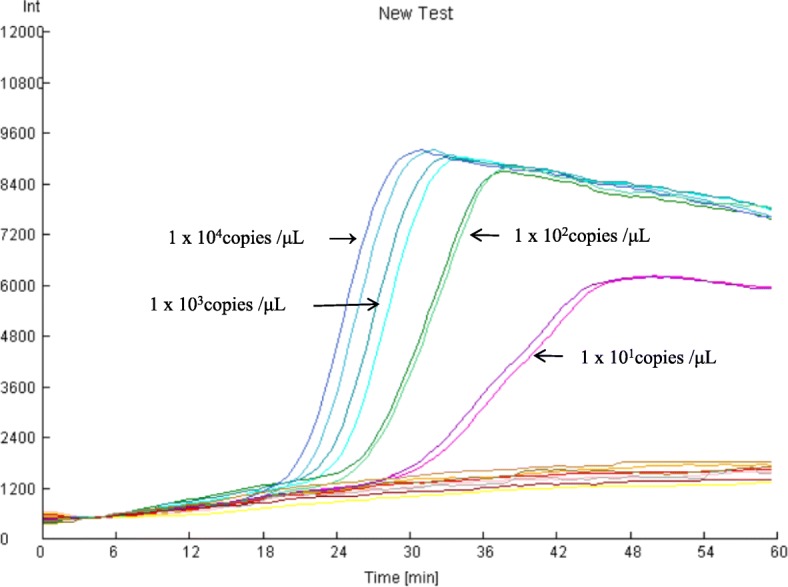
Sensitivity of the LAMP assay for PCV3. Ten-fold serial dilutions of plasmids containing a cloned fragment of the PCV3 capsid gene were used for the assay. Negative control, H_2_O. All reactions were performed in duplicate

Ten-fold serial dilutions of plasmid DNA were used as templates in the assay. The detection limit of the assay was 1 × 10^1^ copies/μL per reaction (Fig. 1). These results indicated the real-time LAMP assay could be used as a sensitive diagnostic test for PCV3.

**Fig. 2:**
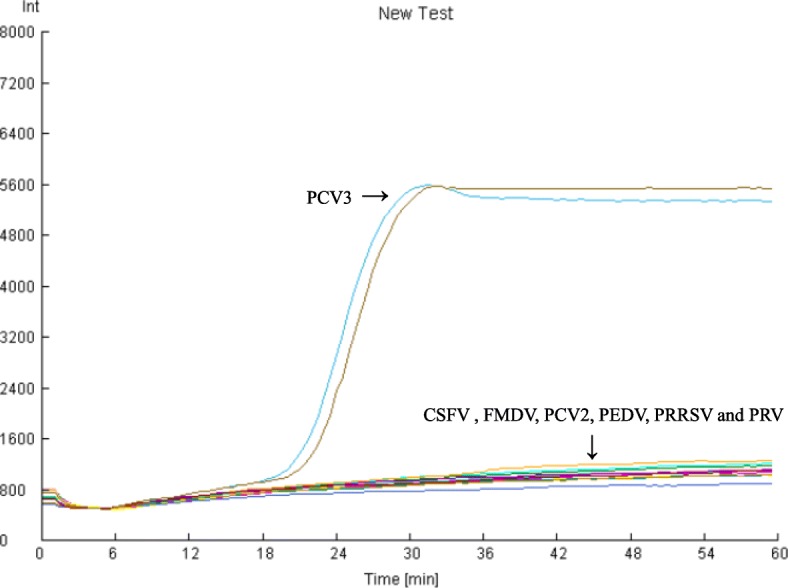
Specificity of the LAMP assay. DNA from PCV2, PRV and cDNA of other swine viral pathogens including FMDV, PEDV, CSFV and PRRSV was tested in the LAMP assay. H_2_O was used as template for mock samples. All reactions were performed in duplicate

The specificity of the reactions was identified using DNA templates of PCV2, PCV3, PRV and other swine viral cDNA samples including those of CSFV, FMDV, PEDV, and PRRSV. The experiment was performed in duplicate. All reactions containing PCV3 were positive in the real-time LAMP assay. The other viral samples mentioned above were negative, indicating that the assay was highly specific for PCV3 (Fig. 2).

### Clinical sample testing

The real-time LAMP were performed to test for PCV3 DNA in 203 clinical samples as previously described [10]. The PCV3-positive rate detected by the real-time LAMP was 89.66% (182/203) (Table 1).

**Table 1:** Screening results for 203 clinical samples for real time LAMP, real-time PCR and conventional PCR assay

Molecular technique	Samples							Proportion positive
	Brain	Heart	Liver	Spleen	Lung	Kidney	Serum	
Real time LAMP	24/36	26/29	32/34	29/31	32/34	29/29	10/10	89.66%(182/203)
Real-time PCR	23/36	26/29	29/34	29/31	32/34	27/29	10/10	86.70%(176/203)
Conventional PCR	6/36	11/29	7/34	8/31	9/34	9/29	4/10	26.6%(54/203)

## Discussion

In this study, a novel real-time LAMP assay for detecting PCV3 was developed, and 203 clinical samples were used to validate the field application of the assay. This assay introduces a new method for the laboratory detection of PCV3 that does not rely on expensive equipment and instrumentation. Thus, the real-time LAMP assay established in this study is a valuable alternative for the detection of PCV3.

To the best of our knowledge, this method is the first real-time LAMP assay for the PCV3 detection, which has a detection limit of 1 × 10^1^ copies/μL for PCV3, and is more sensitive than regular PCR. According to previous reports, the limit of detection of PCV3 was 1.73 × 10^2^ copies/μL and 10^2^ copies/μL with a SYBR Green-based real-time quantitative PCR assay and a TaqMan-based real-time PCR assay, respectively [9, 10]. In this real-time LAMP study, the detection limit was only approximately 1 × 10^1^ copies/μL. Both real-time LAMP and conventional PCR [1] were performed to demonstrate the presence of PCV3 DNA in samples as previously described [10]. The PCV3-positive rates detected by conventional PCR, real-time quantitative PCR and real-time LAMP were 26.60% (54/203) [10], 86.70% (176/203) [10], and 89.66% (182/203), respectively. Furthermore, the samples that were positive for PCV3 by the real-time LAMP assay but negative by the conventional PCR assay were confirmed by the nested PCR (S1) [14]. Positive PCR products were confirmed by sequencing (S2). These results demonstrate that the real-time LAMP assay was sufficient, accurate and reliable as a molecular diagnostic test for the detection of PCV3.

## Conclusion

A novel real-time LAMP assay was developed in this study for the detection of PCV3 nucleic acids. This PCV3 real time LAMP assay was highly specific, sensitive and reliable. This assay will be useful for the diagnosis and epidemiological and pathogenesis studies of PCV3 infections.

## Methods

### Virus strains and clinical samples

Clinical samples including 36 brains, 29 hearts, 34 livers, 31 spleens, 34 lungs, 29 kidneys and 10 sera from pigs from a previous experiment that were stored at Jingyun Ma’s lab (South China Agricultural University, Guangzhou, China) were subjected to a real-time LAMP assay [10]. Six porcine viruses were used for specificity testing of PCV3. These viruses were classical swine fever virus (CSFV) C strain, foot-and-mouth disease virus (FMDV), porcine circovirus 2 (PCV2) LG vaccine strain, porcine epidemic diarrhoea virus (PEDV), porcine respiratory and reproductive syndrome virus (PRRSV), and pseudorabies virus (PRV) [13, 15].

### Nucleic acid extraction

Frozen field samples were homogenized for 30 s with a tissue homogenizer in phosphate-buffered saline (PBS) to prepare samples for extraction procedures. Viral DNA and RNA were extracted from the samples using a TGUide Virus DNA/RNA Kit and T-Guide instrument according to the manufacturer’s instructions (Tiangen, Beijing, China). The RNA viruses were treated with a PrimeScript™ RT reagent Kit with gDNA Eraser kit (Takara, China) according to the manufacturer’s instructions.

### Design of primers for real-time LAMP

Real-time LAMP primers targeting on the PCV3 cap gene (accession number KY418606.1) were designed using the program http://primerexplorer.jp and were synthesized by Sangon (Sangon Biotech, Shanghai, China). The primers included two inner primers (PCV3-FIP and PCV3-BIP) and two outer primers (PCV3 -F3 and PCV3-B3) (Table 2).

**Table 2:** Oligonucleotide primers used in this study

Name	Sequence((5′–3′) and Position
PCV3-B3	GCAGTGCTCCCCATTGAAC (1425–1443)
PCV3-F3	ACACTTGGCTCCAAGACGA (1610–1628)
PCV3-BIP	GAACTACCAGCGCTCACCCAG (1503–1520)-GGTGGGGTCATATGTGTTGA (1444–1463)
PCV3-FIP	TGGGGGTGAAGTAACGGCTGT (1539–1559)-TGCGGAAAGTTCCACTCGTA (1584–1603)
PCV3-LB	TTTCTCCAGACCCACCCCATG (1466–1486)

### Construction of plasmids containing the cap gene of PCV3

The target fragment was amplified using primers PCV3-F3 and PCV3-B3 and cloned into the pMD-18 T vector (Takara, China), which was transformed into *Escherichia coli* competent cells according to the manufacturer’s instructions (DH5α, Tiangen, Beijing, China). The plasmid containing the PCV3 cap gene (pMD-18 T-cap) was purified using a commercial kit (Tiangen, Beijing, China). The concentration of the plasmid sample was determined by measuring the absorbance at 260 nm, using a NanoDrop Lite spectrophotometer (Thermo Scientific, USA), and the copy number of the cloned gene was quantified as follows [16]: [copies/μL = concentration of plasmid (g/μL)/[(plasmid length× 660) × (6.022 × 10^23^)].

### Development of real-time LAMP assay

The real-time LAMP reaction mixture contained template DNA or cDNA, inner primers (FIP and BIP) at 1.6 mM, outer primers (F3 and B3) at 0.2 mM, loop backward primers (LB) at 0.8 mM, 1 μL of Bst DNA polymerase (M5038 L, New England Biolabs, MA, USA), betaine at 0.8 M (BCBS087V, Sigma St. Louis, MO USA), dNTPs at 1.4 mM, 2.5 μL of amplification buffer (B0537S, New England Biolabs), 8 mM of MgSO_4_ and SYTO-9 green fluorescent nucleic acid stain at 0.1 mM (S34854, Invitrogen, Carlsbad, CA, USA). Double-distilled water was used as a negative control. The mixture was incubated at 63 °C for 60 min and then heated at 80 °C for 10 min to terminate the reaction. Finally the results were detected using a Thermostatic Fluorescence Detector (DEAOU-308C, Diao, Guangzhou, China) and are shown by the graph. Specificity of the method was determined using DNA templates of PCV2, PCV3, PRV and other swine viral cDNA samples from CSFV, FMDV, PEDV, and PRRSV. Ten-fold serial dilutions of pMD-18 T-cap were used to calculate the analytical sensitivity. The method was further evaluated using 203 clinical samples [10]. H_2_O was used as template for mock samples. All reactions were performed in duplicate.

## Data Availability

All data generated or analyzed during the study are included in this published article.

## Abbreviations

CSFV: Classical swine fever virus
FMDV: Foot-and-mouth disease virus
PCV2: Porcine circovirus 2
PCV3: Porcine circovirus type 3
PDNS: Porcine dermatitis and nephropathy syndrome
PEDV: Porcine epidemic diarrhoea virus
PRRSV: Porcine respiratory and reproductive syndrome virus
PRV: Pseudorabies virus

## References

[1] Palinski R, Pineyro P, Shang PC, Yuan FF, Guo R, Fang Y, Byers E, Hause BM. A novel porcine circovirus distantly related to known circoviruses is associated with porcine dermatitis and nephropathy syndrome and reproductive failure. J Virol. 2017;91. doi: 10.1128/JVI.01879-16. 10.1128/JVI.01879-16 PMC5165205 27795441

[2] Ku X, Chen F, Li P, Wang Y, Yu X, Fan S, Qian P, Wu M, He Q (2017). Identification and genetic characterization of porcine circovirus type 3 in China. Transbound Emerg Dis.

[3] Kwon T, Yoo SJ, Park CK, Lyoo YS (2017). Prevalence of novel porcine circovirus 3 in Korean pig populations. Vet Microbiol.

[4] Stadejek T, Wozniak A, Milek D, Biernacka K (2017). First detection of porcine circovirus type 3 on commercial pig farms in Poland. Transbound Emerg Dis.

[5] Tochetto C, Lima DA, Varela APM, Loiko MR, Paim WP, Scheffer CM, Herpich JI, Cerva C, Schmitd C, Cibulski SP (2018). Full-genome sequence of porcine circovirus type 3 recovered from serum of sows with stillbirths in Brazil. Transbound Emerg Dis.

[6] Li Gairu, He Wanting, Zhu Henan, Bi Yuhai, Wang Ruyi, Xing Gang, Zhang Cheng, Zhou Jiyong, Yuen Kwok-Yung, Gao George F., Su Shuo (2018). Origin, Genetic Diversity, and Evolutionary Dynamics of Novel Porcine Circovirus 3. Advanced Science.

[7] Zhai SL, Zhou X, Zhang H, Hause BM, Lin T, Liu R, Chen QL, Wei WK, Lv DH, Wen XH (2017). Comparative epidemiology of porcine circovirus type 3 in pigs with different clinical presentations. Virol J.

[8] Kim HR, Park YR, Lim DR, Park MJ, Park JY, Kim SH, Lee KK, Lyoo YS, Park CK (2017). Multiplex real-time polymerase chain reaction for the differential detection of porcine circovirus 2 and 3. J Virol Methods.

[9] Wang J, Zhang Y, Wang J, Liu L, Pang X, Yuan W (2017). Development of a TaqMan-based real-time PCR assay for the specific detection of porcine circovirus 3. J Virol Methods.

[10] Chen GH, Tang XY, Sun Y, Zhou L, Li D, Bai Y, Mai KJ, Li YY, Wu QW, Ma JY (2018). Development of a SYBR green-based real-time quantitative PCR assay to detect PCV3 in pigs. J Virol Methods.

[11] Oscorbin IP, Belousova EA, Zakabunin AI, Boyarskikh UA, Filipenko ML (2016). Comparison of fluorescent intercalating dyes for quantitative loop-mediated isothermal amplification (qLAMP). Biotechniques.

[12] Yu X, Shi L, Lv X, Yao W, Cao M, Yu H, Wang X, Zheng S (2015). Development of a real-time reverse transcription loop-mediated isothermal amplification method for the rapid detection of porcine epidemic diarrhea virus. Virol J.

[13] Wang H, Cong F, Zeng F, Lian Y, Liu X, Luo M, Guo P, Ma J (2018). Development of a real time reverse transcription loop-mediated isothermal amplification method (RT-LAMP) for detection of a novel swine acute diarrhea syndrome coronavirus (SADS-CoV). J Virol Methods.

[14] Yongning Y, Yijia R, Luchang S (2019). Establishment and application of nested PCR for detection of porcine circovirus type 3. Heilongjiang Anim Sci Vet Med.

[15] Zeng F, Cong F, Liu X, Lian Y, Wu M, Xiao L, Yuan W, Huang R, Ma J, Guo P, Luo M (2018). Development of a real time loop-mediated isothermal amplification method for detection of Senecavirus a. J Virol Methods.

[16] Parida M, Shukla J, Sharma S, Ranghia Santhosh S, Ravi V, Mani R, Thomas M, Khare S, Rai A, Kant Ratho R (2011). Development and evaluation of reverse transcription loop-mediated isothermal amplification assay for rapid and real-time detection of the swine-origin influenza a H1N1 virus. J Mol Diagn.

